# Starch from Unripe Apples (*Malus domestica* Borkh) as an Alternative for Application in the Food Industry

**DOI:** 10.3390/molecules29081707

**Published:** 2024-04-10

**Authors:** Dorota Gumul, Jarosław Korus, Magdalena Orczykowska, Justyna Rosicka-Kaczmarek, Joanna Oracz, Anna Areczuk

**Affiliations:** 1 Department of Carbohydrate Technology and Cereal Processing, University of Agriculture in Krakow, Balicka 122 Str., 30-149 Krakow, Poland; rrkorus@cyf-kr.edu.pl (J.K.); anna.areczuk@urk.edu.pl (A.A.); 2 Department of Chemical Engineering, Faculty of Process and Environmental Engineering, Lodz University of Technology, Wolczanska 213 Str., 90-924 Lodz, Poland; magdalena.orczykowska@p.lodz.pl; 3 Institute of Food Technology and Analysis, Faculty of Biotechnology and Food Sciences, Lodz University of Technology, 2/22 Stefanowskiego Str., 90-537 Lodz, Poland; justyna.rosicka-kaczmarek@p.lodz.pl

**Keywords:** *Malus domestica*, immature apple, starch, physicochemical characteristic, thermal properties, rheological properties, molecular weight

## Abstract

This study investigated the properties of starch isolated from the unripe fruit of two apple cultivars (*Malus domestica* Borkh) grown in southern Poland (Central Europe). The chemical composition of both starches, molecular mass, their granulation, thermal characteristics, swelling characteristics, and rheological characteristics were studied. The starches differed significantly in ash, phosphorus, and protein content. The water-binding capacity at temperatures of 25–65 °C was similar, while differences of 20% appeared at higher temperatures. In contrast, a significant difference was found in the solubility of the two starches in the temperature range of 25–75 °C. The study showed that apple starches have a relatively low tendency to retrograde, with the enthalpy of gelatinization for starch from the Oliwka variety being 40% higher than that from the Pyros variety. However, the starches differed in the hardness of the gels formed, i.e., one variety formed soft gels with an internal structure resistant to external forces, while the other formed hard gels.

## 1. Introduction

Starch is the main biopolymer that can be found in tubers, cereal grains, seeds, roots, stem core and rhizomes, and fruits. Starch is a generally recognized as safe (GRAS) renewable polysaccharide that is known to be non-allergenic, has good biocompatibility and biodegradability, and is inexpensive [1,2]. This polysaccharide is of great importance to the industry [1,3], and thus a comprehensive understanding of its physicochemical properties is very important. In addition, starch is one of nature’s most abundant plant carbohydrate reserves and is also found in some algae. Unlike glycogen, which is similar in structure and found in animals, starch naturally occurs in the form of water-insoluble granules that undergo a decomposition known as gelatinization when heated in an aqueous environment [4]. The properties of starches from different plants vary, sometimes significantly, among themselves [2,3,5,6]. Due to its widespread occurrence, ease of isolation from plant material, maturational variation in properties, and susceptibility to chemical, physical, and enzymatic modification, starch is widely used in various industries—in food and non-food applications [4,7]. However, in order for starch to be used as a food ingredient, its chemical composition, molecular weight, granularity, physicochemical properties such as solubility, water-binding capacity, gelatinization, retrogradation, and rheological properties must be comprehensively examined, since the aforementioned characteristics of starch will determine the further use of this polysaccharide and its modifications in industry.

A wide range of applications in various industries have found starch to be derived mainly from corn, wheat, potatoes, and cassava. For this reason, the physical and chemical properties of starches obtained from these raw materials are already very well known [3,6]. Fruits are not among the richest sources of this polymer; however, the growing demand for starch is driving the search for new ways of obtaining this raw material. Also, starch isolated from different fruits differs in structure, chemical composition, and physical properties, thereby making it possible to obtain raw materials for various food and non-food applications [8]. The most apples in the world are produced by China, the European Union (in which Poland is the largest producer), the USA, and Turkey [9]. Apple production in Poland was almost 4.1 million tons in the 2015/2016 season. It is expected that over the next few years, the harvest could reach 4.5 to even 5.0 million tons in the case of favorable weather conditions. The overproduction of apples is due to various reasons, including a general increase in yields or shrinking markets. These factors foster a reduction in the price of apples and stimulate a search for new possibilities to use table apples in industrial production or to use them as animal feed. Therefore, it is crucial to explore new alternative applications for these fruits [10,11,12]. The main industrial uses of apples are the production of juices, wines, cider, distilled spirits, and vinegar [9,13,14]. Such a large overproduction of apples in Poland makes it vital to seek alternative applications than those mentioned above. Although the eventual production of starch from apples will not completely solve the problem, it could be one promising direction for tackling the surplus of these fruits. Therefore, in this work, apples were used as a source of starch, but at an incomplete maturity stage. During fruit ripening, the starch content decreases as it is converted to sugars [15]. In addition, as demonstrated by Gambuś et al. [16] for wheat, rye, and barley and Liu et al. [17] for potatoes, the properties of starch change with ripening. Therefore, it is likely that starch from unripe apples differs from starch from ripe fruit. At the same time, in the case of ripe apples, starch extraction is pointless, because as mentioned earlier, starch is converted to simple sugars during ripening. For the above reasons, it was decided to study the possibility of isolation and physicochemical properties of starch from unripe apples (*Malus domestica* Borkh). The feasibility study focused on the use of unripe apples as an alternative source of starch. According to many authors [18,19,20,21], the main factors determining the physical and chemical properties of starch are the region in which apples are grown, followed by climatic, soil, agrotechnical, and varietal conditions.

Thus, the purpose of this work was to isolate starch from unripe apples grown in southern Poland (Central Europe) and to analyze their granularity, non-carbohydrate substance content, weight-average molecular weight, and physicochemical properties, with particular emphasis on the rheological properties.

## 2. Results and Discussion

### 2.1. Chemical Composition

The amylose content plays a main role in the physicochemical properties of starch [22]. The starches hardly differed in terms of amylose content (Table 1). The amylose content was close to its lower limits in wheat, oat, or rice starches [23]. Stevenson et al. [18], on the other hand, showed that the amylose content in apple starch reaches up to 40–48% (apparent amylose) and 26–29% absolute amylose (after counting the iodine affinity of amylopectin). A study by Doerflinger et al. [24] showed a decrease in the amylose content of apple starch with increased ripening times. Therefore, it is difficult to explain the discrepancies between the results of the present study on starch from unripe fruit and those of other authors, as there is no information available on the exact stage of ripening at which the apples were analyzed.

The amount of amylose and amylopectin is about 98–99% in the dry weight of potato starch granules. The remaining components, which can include lipids, phosphoric acid esters, ash, and protein, are regarded as non-carbohydrate components of starch granules [25]. A certain amount of non-carbohydrate substances, such as protein, fat, ash, and phosphorus, incorporated into starch granules during their isolation can affect the physicochemical properties of starch, such as the water-binding capacity, solubility, and gelatinization [2,3]. Therefore, the determination of these substances is very important. The amount of protein in the tested starches was about 0.5%, with about 10% less protein in starch from the Pyros (SP) variety than in starch from the Oliwka (SO) variety. Also, a higher amount of ash and phosphorus was observed in SO than in SP by 49% and 17%, respectively. For lipids, no differences were observed between the two types of starch (Table 1). Considering the weight-average starch molecular weights of the two varieties tested, it was shown that they did not differ. To our knowledge, no one has so far determined the weight-average molecular weight of starch from unripe apples; only Stevenson et al. [18] determined the weight-average molecular weight of amylopectin from starch extracted from unripe apples. It ranged from 4.63 to 11.10 × 10^8^ g/mol, with the differences due to the variety of apples from which the starch was isolated. Both starches tested in this study contained about 15% amylose, meaning that about 85% was amylopectin. Therefore, this fraction had the greatest impact on the weight-average molecular weight of starch; hence, the order of 10^8^ g/mol was the same as in the studies of Stevenson et al. [18].

Comparing the starch isolated from potatoes and apples, it was found that the amount of protein in potato starch was 10 times lower than in apple starch. Indeed, for potato starch, the amount of protein was in the range of 0.05–0.1%, while the amount of lipids was in the range of 0.05–0.1% [26]. In contrast, Lizarazo et al. [27] estimated the amount of protein in potato starch in the range of 0.42–0.43%, and in a study by Gunaratne and Hoover [28], the level of protein was determined in the range of 0–0.05. In a study by Heo et al. [29], they reported a protein content of 0.5% in potato starch and a lipid content of about 0.31%. Comparing the protein content of potato starch in the study of Heo et al. [29] with the results obtained in this work for apple starch, it can be noted that the protein content of both types of starch was similar and the amount of lipids was four times lower in potato starch than in apple starch. The amount of lipids in legume starch is at the level of 0.05–0.19, and the ash content ranges from 0.01 to 0.085% [30]. It was shown that the amount of lipids in apple starch was 4 to 12 times higher than in potato starch and 6 times higher than in legume starch [26]. In the case of corn starch, the most popular starch in the world, the protein content was 0.73% and the level of lipids was 0.58% [31]. Comparing the protein content of the apple starches discussed in this work with corn starch [31], it was noted that a higher content of this component by about 30% was found in corn starch than in apple starch. The lipid content was 35% lower in the apple starches analyzed in this work than in the corn starches studied by Yousif et al. [31].

Phosphorus is a very important non-carbohydrate component of starch granules, as it determines many functional properties of starch. In potato starch, phosphorus is 0.6% [26] or 0.04–0.12% [32]. Similar levels of phosphorus have been reported in apple starches (Table 1). In a study by Park and Chung [33] on apple varieties grown in Korea, the amount of phosphorus in starch was 0.013%. In the case of ash, its content was determined by Kaur et al. [19] in the range of 0.06–0.43% in potato starches, while in a study by Lizarazo et al. [27], the amount was in the range of 0.38–0.87%, and Heo et al. [29] determined the ash content as 0.2%. In corn starch, the amount of ash oscillated around 0.78% [31], which was a similar value to the ash content in the apple starch studied in this work (Table 1). The ash content of potato starch is determined by the mineral content of the soil in which the tuber grows [27]. According to Gunaratne and Hoover [28], the final ash content of starch is largely influenced by phosphorus and calcium. The ash content in apple starches is similar to its average level in potato starches.

According to Alcazar-Alay and Meireles [34], the chemical composition of starch, i.e., protein, lipids, ash, and phosphorus content (non-carbohydrate components), is affected by the potato variety from which it originated, the climatic, soil, and agrotechnical conditions in which the potato tuber was grown, and, from which the starch was extracted. Similarly, the amylose content of potato starch, according to Lizarazo et al. [27], largely depends on the differences in amylose determination methodology as well as climatic and soil conditions.

### 2.2. SEM and Granularity

**Figure 1:**
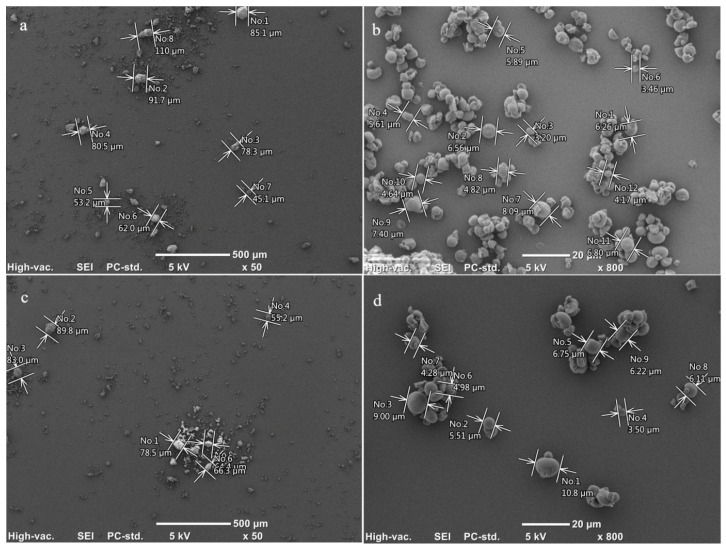
Scanning electron microphotographs of SO (**a**,**b**) and SP (**c**,**d**).

Scanning electron micrographs of the two apple starches showed similar granule morphology and size distribution, with diameters ranging from 3.5 to 11 µm for SP and from 3.2 to 8.09 µm for SO (Figure 1). The sizes of the apple starch granules analyzed in this work were similar to the average size of starch granules (9.21 µm) found in apple juice [35]. The granules were spherical or dome-shaped and split, and they were likely synthesized as complex starch. According to Stevenson et al. [13], apple starch ranges in size from 2 to 12 µm. In the case of a study by Park and Cheung [33] regarding the starch of apples from Korea, the average size of starch grains oscillated around a value of 7.5 µm. Apple starch is similar to pumpkin fruit starch and starch from kiwifruit, while it is different from starch from mango kernels (oval to elliptical shapes; average 19.32 µm), starch from the banana peel (an oval and irregular shape with eccentric hilum—average 17 µm), annatto seeds (spherical-like shape; average 0.8 µm), and starch from potato (spherical or spherical; on average, 2–110 µm) [36,37]. The distribution of apple starch is unimodal (Figure 1) in contrast to the bimodal distribution of starch from cereals (small granules under 10 µm and large granules above this value) and starch from litchi seeds (bimodal distribution of small granules between 3.0 and 4.22 µm and large in the range 7.8–10.2 µm–round and oval shape) [8,27]. At the same time, it was observed that apple starch can occur as single granules or form large conglomerates (clusters) (Figure 1). Considering the granularity of the analyzed apple starches, it was shown that 67.3 to 75.2% of the starch consisted of small starch granules. Starch with dimensions of 30–70 μm and above 70 μm is considered here as conglomerates (sets of starch granules connected with each other), which in SO account for about 32% while in SP account for 24%. This nature of apple starch is confirmed by SEM images and granularity measurements from a laser particle size analyzer (Table 1, Figure 1).

### 2.3. Swelling Characteristics

The swelling properties of starch, especially the water-binding capacity (WBC), depend on the amylopectin content [34,38], as well as on the molecular weight of this polymer and the shape and conformation of the molecules, which build up the crystalline part of the starch granules [38]. Amylose, on the other hand, is considered a starch polymer that reduces its swelling due to the fact that it could strengthen the internal structure of the starch granule [34]. According to other authors [34,38], amylose acts as a “thinner” and an inhibitor of starch swelling. Considering water-binding capacity in general, it can be concluded that starch from the apple varieties analyzed had an identical WBC in the temperature range of 25–60 °C, which was not surprising as they had similar amylose contents. In contrast, differences in the WBC were observed at higher temperatures, where, at 75 and 95 °C, the WBC value for SO was approximately 20% higher than for SP. (Table 2). It should also be noted that the water-binding capacity of starch is also affected by the phosphorus content [38] as well as the way that it is arranged on adjacent amylopectin chains [39]. Such large WBC differences between two analyzed starches at 75 and 95 °C may be explained by the higher amount of phosphorus (by 17%) in SO than in SP (Table 2). The water-binding capacity and solubility (S) are also influenced by the granularity of the starch since, according to the aforementioned authors, large starch granules are characterized by a higher value of the aforementioned parameters. In order to consider the effect of granularity on the water-binding capacity and solubility, the percentage of starch granules between 30 and 70 μm and >70 μm was taken into account, considering them as conglomerates of apple starch granules (Table 1). It was found that within the tested apple starches, the SO had a higher proportion of conglomerates, which contributed to a higher water-binding capacity at 75–95 °C. Considering the solubility of SO, it was found that this sample showed significantly higher solubility (two times higher) over the entire range of temperatures from 25 to 75 °C than SP. This is because SO had a higher number of tightly bound granules. The exception was the solubility of SO at 95 °C because it was identical to the starch from SP. Another factor affecting the water-binding capacity of starches is the lipid content. According to Swinkels [40], due to the low levels of lipids in potato starches, lipids have little or no effect on water absorption. This contrasts with cereal starches, where phosphorus is in the form of phospholipids, which significantly reduce the swelling of the granules, especially in starches with a high lipid content, such as oat starch [41]. In the case of corn starch, the lipids occur as free fatty acids. It should be noted that lipids that are present on the surface of starch granules can combine with amylose to form amylose–lipid complexes (ALCs), which contribute to some phenomena that are undesirable from a technological point of view. For example, in the production of hydrolysates, ALCs reduce the hydrophilicity, swelling, and dissolving of starch, which delays its gelatinization and prevents enzymatic hydrolysis [42]. The amount of lipids in the two apple starches considered in this work was identical at 1.20%, meaning that they had no effect on the swelling properties of the starch (Table 1 and Table 2). On the other hand, the high amount of lipids in apple starches compared to potato starches, where they represent about 0.1%, could lead to a strong reduction of WBC and S apple starches in comparison to potato starches. Pietrzyk et al. [43] studied the water-binding capacity of potato starch at 60 °C and 70 °C, obtaining results of 1.08 g/g and 25.54 g/g, respectively. Heo et al. [29] determined the WBC at 50, 60, 70, and 80 °C, where these values were 1.3, 3.34, 18.24, and 32.70 g/g, respectively. According to Khan et al. [44], the solubility percentages at 60, 70, 80, and 90 °C were 7%, 12%, 12%, and 17%, respectively.

Accordingly, the swelling parameters (S and WBC) were higher for potato starch than for apple starch, which can be explained by the high amount of lipids, 4 to 12 times higher in apple starch than in potato starch, corresponding to a reduction in the S and WBC of apple starch (Table 2). Thus, when considering the water-binding capacity and solubility of starch, it was concluded that many factors should be taken into account, and none of them should be overlooked.

### 2.4. Thermal Characteristics Performed by Differential Scanning Calorimetry (DSC)

The differences in the thermal characteristics of the tested starches were not great, but in most cases, they were statistically significant. The greatest differences were found for Δ*H* (Table 2). The Δ*H* value was about 40% higher for SO than for SP samples. On the other hand, the values of gelatinization enthalpy of both starches were lower than reported for corn, wheat, potato starches [45,46], and apple starch [36], while they were similar to wheat starches tested by Majzoobi et al. [47]. Liu et al. [46] indicated that the gelatinization enthalpy of potato starch increased slightly with an increasing cultivation time. Similarly, the relatively low gelatinization enthalpy of the studied apple starches might be influenced by their short ripening period. As indicated by Majzoobi et al. [47], Δ*H* may reflect the degree of amylopectin crystallinity associated with the distribution of short amylopectin chains or the loss of the double-helix structure during gelatinization. Low enthalpy values are related to the lower crystallinity of starch from the Pyros variety (Table 2) or a lower number of hydrogen bonds present in its granules [48]. However, the other parameters of the gelatinization characteristics of the starches (*To*, *Tp*, and *Tc*), despite the significance of the differences, do not support this supposition. Among others, the temperature of gelatinization depends on the structure of amylopectin, the composition of the starch (content of amylose, amylopectin, and phosphorus), the structure of the starch granules, and the proportion of amorphous and crystalline regions [47]. In the present study, it was found that one of the starches tested (SP) had a slightly higher amylose content (and thus probably a lower amylopectin content). According to Sikora et al. [49], the value of *To* depends on the amount of amylose, i.e., a higher level of this polymer results in higher values of *To*. This was also confirmed in this work, where the higher onset gelatinization temperature was observed in SP samples, i.e., those with a higher amylose content.

Chen et al. [50], who studied starches of different botanical origins (cereal and tuber), found that the temperature of gelatinization is influenced by the length of the chains that build up amylopectin branches and their distribution. They also unequivocally showed that potato starch has the lowest onset gelatinization temperature due to the high phosphorus content of potato starch compared to starches of other botanical origins, such as cereals. This is confirmed by the results of this work, where SO had a higher phosphorus content than SP, which was reflected by lower *To* (Table 1 and Table 2). The differences in *Tc*-*To* values, on the other hand, were not statistically significant (Table 2). The values of these parameters were lower than in the apple starches studied by Stevenson et al. [18]. The differences may be due to varietal factors, growing conditions, or maturity; e.g., the average fruit weight of the apples studied by Stevenson et al. [18] was 100–159 g, depending on the variety, while in the present study, it was only 35–40 g.

### 2.5. Retrogradation

Starches from unripe apples showed a low retrogradation susceptibility, as determined by two methods. The first method is based on the loss of the ability of starch polymers to form a complex with iodine [51]. The second method relates to the increase in the starch retrogradation (recrystallization) enthalpy Δ*H_r_* of the paste stored for a certain period of time during the heating in a differential scanning calorimeter (DSC). The melting of crystals formed during the recrystallization of the amylose and amylopectin or co-crystallization of both polymers was analyzed as an indicator of those changes because of their reversible character in the temperature region close to 100 °C [52]. The enthalpy of retrogradation for the SO sample was found to be a little higher than that of the SP sample after 24, 48, and 72 h of storing the paste at 8 °C (Table 3). Thus, it can be suggested that slightly more intensive recrystallization may have occurred in the SO sample compared to the SP sample. The value of the degree of retrogradation of the 1% aqueous starch pastes was determined by measuring the absorbance of the iodine–starch complex and is shown in Table 3. During storage of the starch pastes, retrogradation occurred in all of them, as evidenced by increasing values of the calculated degree of retrogradation. The degree of retrogradation of 1% aqueous starch pastes made from the SO sample was slightly higher compared to pastes made with the SP sample. The trend was observed when pastes were stored both above 0 °C (8 and 20 °C) and in samples subjected to freezing –20 °C. It should be noted, however, that both the starch from the Oliwka (SO) apple variety and the starch of the Pyros (SP) apple variety had a low tendency to retrograde, as determined by two independent methods.

This low retrogradation susceptibility of the above-mentioned starches should be explained by the low amylose content and low molecular weight of these starches (Table 1). It should also be mentioned that during the retrogradation process, amylose is recrystallized first, while amylopectin is recrystallized later in a continuous and prolonged manner. Thus, in the first stage, lasting up to 48 h after the preparation of pastes, the short-term retrogradation of amylose was dominant, while further changes should be attributed to the retrogradation of amylopectin [53]. The reason for the short-term retrogradation was an increase in the cross-linking of starch molecules through the formation of hydrogen bridges between adjacent amylose chains. This resulted in an increase in the crystalline regions of starch and the formation of larger aggregates [53,54,55]. The degree of retrogradation of amylose, which is responsible for short-term retrogradation, depends on a number of factors, including the ratio of amylose to amylopectin, the length of the chains, the concentration of the solution, and also the presence of starch-associated substances [56]. According to Kaur et al. [57], starch retrogradation could also be influenced by the arrangement of starch chains in the crystalline and amorphous regions of the starch granules, which in turn can affect the degree of grain destruction during starch pasting. Starch retrogradation is a phenomenon that occurs during the storage of starch products and is one of the main factors causing the deterioration of food quality. Retrogradation may be largely responsible for the staling of baked goods [52]. Therefore, it is important to obtain starches with a low tendency to retrograde. Based on the retrogradation results, it can be suggested that starch from unripe apples that has a low tendency to retrograde is more digestible than typical starches used in the food industry. Starch digestibility decreases as the amount of amylose increases [58,59]. In this work, it was shown that the amylose content is low, at 15 to 16%. According to Wei et al. [60], starch from unripe bananas shows an enzyme-resistant starch fraction content of 48.9%. Espinosa-Solis et al. [58] found that the elevated degree of amylolytic resistance is associated with a high amylose content in banana starch (36%) and mango starch (31%). Thus, the low amylose content presented in this work in starches from unripe apples (Table 1) should contribute to its increased digestibility.

### 2.6. Assessment of Viscous Properties

**Figure 2:**
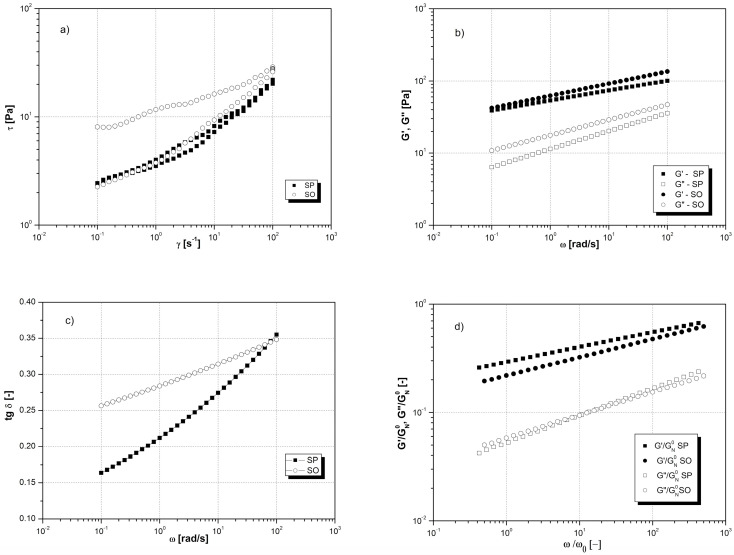
Rheological properties of apple starch pastes from Pyros and Oliwka varieties: (**a**) flow curves; (**b**) mechanical spectra; (**c**) the tangent of the loss angle *δ* as a function of the oscillation frequency *ω*; and (**d**) reduced curves of mechanical spectra.

The analysis of the obtained experimental data allowed us to conclude that the tested samples of apple starch are media showing the phenomenon of thixotropy in the form of a characteristic hysteresis loop (Figure 2a). The areas of the hysteresis loop of the two tested apple starch samples differed significantly in size. Thus, for the SP apple starch sample, the hysteresis loop area was 104.69 Pa·s^−1^, while for the SO apple starch, it was 451.79 Pa·s^−1^. The area of the hysteresis loop for apple starch SO was thus more than four times greater than the area of the hysteresis loop of apple starch SP. Consequently, this means that the SO apple starch structure is much more susceptible to shear failure than the SP apple starch structure.

Table 4 shows the flow curves obtained from rheometric measurements at increasing and decreasing shear rates. Of the three rheological models used, the Herschel–Bulkley model described the results obtained with the highest accuracy, which is directly indicated by the statistical criteria in the form of the highest R^2^ values (at the level of 0.998) and at the same time the lowest RMSE values (in the range of 0.432–0.6). Obviously, the tested apple starches should be treated as non-Newtonian media, which are non-linear or non-Bingham viscoplastic fluids. Therefore, the comparative analysis of the tested apple starches has been based solely on the Herschel-Bulkley model.

Apple starch SO is characterized by a definite higher yield point *τ_y_*. This shows that the limit shear stress at which it begins to flow for the flow curve with increasing shear rates is more than 2.5 times higher than for apple starch SP. However, the susceptibility of the structure of this starch (SO) to shear failure does not allow it to re-achieve this value of the yield point after the cessation of shear. This means that after the shear forces cease, SO apple starch begins to flow at much lower values of the shear stress limit, which is only 1.24 Pa and is four times lower than before the shear forces appeared. In this respect, apple starch SP is much more stable, the yield point of which does not change significantly under shear. The values of the limit tangential stress before and after the occurrence of shear forces differ by only 0.115 Pa.

Changes in the value of the characteristic flow index *n* and the consistency coefficient *k* with increasing and decreasing shear rates confirm that SO apple starch does not have an internal structure resistant to shear forces. The value of the parameter *n* after the cessation of shear changes by 58% compared to the value before the occurrence of shear forces, and the value of the consistency coefficient *k* changes twice. The parameters *n* and *k* of the Herschel–Bulkley model also confirm the stability and resistance of the internal structure of SP apple starch to shear damage (Table 4).

### 2.7. Assessment of Viscoelastic Properties

Figure 2b shows the experimental values of the storage modulus *G*′ and the loss modulus *G*″ as a function of the oscillation frequency *ω* obtained for the tested apple starches, i.e., the so-called mechanical spectra of SP and SO starch pastes. In the all-analyzed range of oscillation frequency, both apple starches show the advantage of elastic properties over viscous ones in the form of higher values of the storage modulus *G*′ over the loss modulus *G*″.

The data in Table 5 show the following:

- The total elasticity of the network *G_e_* of gelatinized apple starches SP and SO is comparable, with only a slight advantage—about 4 Pa—for the sample of SO starch paste.
- Equilibrium compliance Je, and thus, the possibility of storing energy, is slightly higher for SP starch paste.
- Quite high values of the viscoelastic modulus of the plateau *G_N_*^0^ show that the tested samples of apple starch are media with a structure that shows the behavior typical of viscoelastic quasi-solids with a strong structure. The values of the *G_N_*^0^ modulus indicate that the cross-linking of the structure is stronger for SO starch. What is more, for this starch, aging processes take place slower over time.
- Plateau compliance *J_N_*^0^ is, however, 30% lower for SO starch than for SP starch, which means that SP starch has such network entanglements that suppress all kinds of long-range configuration rearrangements.
- Newtonian viscosity under steady flow conditions *η*_0_ has a much higher value for apple starch SO, which means that the flow capacity of the set of elements closed with the minimum number of network nodes for this starch is lower than for starch SP, and thus starch paste SO is characterized by greater gel stiffness.
- Cross-linking densities *ω*_0_ of both apple starch pastes are at a comparable level.
- The width of the viscoelastic plateau *L* has a higher value, equal to 5.141, for SO apple starch; therefore, this starch has a higher polydispersity than SP starch.
- The values of the average molecular weights for *M_e_* entanglement and *M_c_* cross-linking have the same order of magnitude and similar values, but with a slight advantage in favor of SP apple starch.
- Apple starch SP also creates a network, with the mesh size *ξ* being 3.5 nm larger than in the case of SO starch.

**Table 5:** Rheological parameters of tested apple starch pastes.

Rheological Parameters	Starch Pastes Sample		Unit
	SO	SP	
*G_e_*	42.185 ± 0.107	38.927 ± 0.083	(Pa)
*J_e_*	0.0237 ± 0.0003	0.0257 ± 0.0005	(1/Pa)
*G_N_* ^0^	216.89 ± 1.631	150.54 ± 0.890	(Pa)
*J_N_* ^0^	0.0046 ± 0.0001	0.0066 ± 0.0003	(1/Pa)
*η*_0_	1082.13 ± 3.213	637.65 ± 1.846	(Pa·s)
*τ_m_*	25.652 ± 0.026	16.381 ± 0.017	(s)
*τ*_0_	4.989 ± 0.090	4.236 ± 0.062	(s)
*ω*_0_	0.200 ± 0.004	0.236 ± 0.006	(1/s)
*L*	5.141 ± 0.085	3.867 ± 0.032	(-)
*M_e_*	1.238 × 10^4^ ± 0.129	1.784 × 10^4^ ± 0.114	(kg/mol)
*M_chem_*	6.366 × 10^4^ ± 0.328	6.899 × 10^4^ ± 0.386	(kg/mol)
*ξ*	27.391 ± 0.019	30.937 ± 0.028	(nm)

Data are presented as the mean value of three replicates ± standard deviation.

The analysis of the discussed rheological parameters shows that SP starch forms a soft, elastic gel with a structure that is quite resistant to external mechanical vibrations. This starch is mainly due to its Newtonian viscosity *η*_0_, which is lower than that of SO starch, and the plateau compliance *J_N_*^0^ is greater than that of SO starch. These last two parameters also indicate that SO starch forms a rather hard gel and, moreover, has a higher polydispersity. As a result, the SP starch structure is more resistant to external forces than the SO starch structure, and it has the ability to suppress mechanical vibrations that could contribute to its disintegration. Therefore, this confirms the results obtained in the controlled shear rate mode, which showed that the internal structure, which is much more resistant to shear damage, is possessed by apple SP starch.

For both apple starches, the tangent of the loss angle ranged from 0.1 to 0.35 (Figure 2c). This indicates that the gelatinized apple starches SP and SO, whose rheological properties were tested at 50 °C, have the characteristics of cross-linked and non-cross-linked amorphous polymers, or biopolymers.

The reduced curves of mechanical spectra *G*′/*G_N_*^0^ = f(*ω*/*ω*_0_) are not subject to superposition, which proves the varied share of elements shaping the elastic properties of these starches and the quite diverse scale of dissipation phenomena in the analyzed range of oscillation frequency (Figure 2d). Full superposition of reduced curves was observed for curves *G*″/*G_N_*^0^ = f(*ω*/*ω*_0_). This proves a very similar type of sticky element shaping the internal structure of apple starch, both SP and SO; moreover, the viscous properties of these starches do not seem to be significantly differentiated.

Both apple starches are media with a structure representing the behavior typical of viscoelastic quasi-solids with a strong structure. While the share of sticky elements shaping the internal structure of these starches is not differentiated, the share of elastic elements has already varied. This is one of the reasons why SP forms a soft and elastic gel with a structure resistant to external forces, in contrast to SO, which tends to form stiff and hard gels. This is most likely due to the fact that the starch isolated from apples of the Pyros variety had a slightly lower amylopectin content. It is noteworthy that the content of amylopectin in both isolated apple starches differed by only 1%. In addition, it should be remembered, however, that it is amylopectin with short branches that is responsible for the crystallinity of starch [34], and the starch crystallinity is based on the double helix formed by amylopectin molecules. Thus, the crystallinity of SP is slightly lower compared to SO. The conducted thermal tests, and especially the gelatinization enthalpy value, which is higher for SO, showed that there is a loss of the molecular order of the double helix formed by amylopectin molecules. As a result, a slightly higher content of amylopectin in SO may lead to instability of its structure and a lack of resistance to external forces. The chemical analysis of starches isolated from both apple varieties revealed that SO contain higher level of phosphorus. According to Alcazar-Alay and Meireles [34], the phosphorus content in starch (determined by the example of potato starch) reflects the pasting enthalpy Δ*H_k_* of this starch. This is due to the presence of phosphorus monoesters linked to short amylopectin chains forming crystalline regions in the starch granules. The phosphate groups “repel” each other and destabilize the crystal structure. Thus, a lower content of phosphorus monoesters combined with a lower content of amylopectin—as in the case of SP—results in increased stabilization of the internal structure and its greater resistance to external forces, which in turn was also shown and confirmed by the conducted rheological research.

In summary, as shown, the physicochemical properties of starch affect its functional properties. Understanding the physicochemical properties will allow the selection of starch with properties suitable for a specific application, for example, to reduce the retrogradation process. In addition, knowledge of the physicochemical properties of starch will enable the precise selection of its source and method of modification to achieve the desired functional properties for a specific application end use. Modified starch can have a number of properties, i.e., stabilizing, carrier, thickening, adhesion, emulsifying, etc. In addition, the research presented here can serve as a starting point for further studies aimed at better understanding the behavior of gels obtained from starches isolated from apples. Here again, the need is recognized for further exploration of possible applications, optimization of the process of obtaining (isolation), and also control of gel production processes.

The results presented in this work show that starches obtained from unripe apples differ in composition, as well as in rheological and thermal properties, from other starches obtained from common raw materials, affecting their potential application in food technology.

## 3. Materials and Methods

### 3.1. Materials

Materials for the study were two varieties of apples: Oliwka and Pyros, which were cultivated at the Experimental Farm of the University of Agriculture in Krakow, Poland (Garlica Murowana, Zielonki). Unripe apples were harvested in May 2021, and their fruits were green and did not exceed 3.5 cm in diameter and weight 35–40 g.

### 3.2. Starch Extraction

A total of 1 kg of stalkless apples was poured into 1.5 L of distilled water and crushed with a Zelmer ZHM0861X mixer (Warsaw, Poland). Then, 1 g of xylanase (X2753, Sigma, St. Louis, MO, USA) was added, and the whole mixture was mixed with an RW 20 mechanical stirrer (IKA, Staufen, Germany) for 2 h. The sample was then filtered through a 315 µm mesh sieve, followed by centrifugation for 15 min at 4500× *g*. After removing the top layer of impurities, 500 mL of distilled water was added to the sample, and, after mixing, the sample was centrifuged as above. The operation was repeated 3 times. The purified starch was air-dried. The dry starch was ground in a ball mill (Pulverisette 2, Fritsch, Idar-Oberstein, Germany) and sieved through a 200 µm mesh sieve.

### 3.3. Scanning Electron Microscopy

Microscope observations were performed in accordance with the method previously reported by Rosicka-Kaczmarek et al. [61]. Microscopic images were recorded using a Jeol-JCM-6000 scanning electron microscope (Akishima, Japan). The examined samples were sputter-coated with gold under vacuum (without any noble gas), and images were recorded at various acceleration potentials ranging from 5 kV to 10 kV.

### 3.4. Granule Diameter and Particle Size Distribution

Native starch was analyzed using laser particle size analyzer Analysette 22 NeXT (Fritsch, Idar-Oberstein, Germany) instruments. A starch sample (0.1 g) was weighed and dispersed in deionized water using a vortex mixer (10 s). The measurement was performed according to the standard operating procedure.

### 3.5. Amylose Content

To determine the apparent amylose content (blue complex with iodine), the measurement (run in triplicate) was performed spectrophotometrically. A total of 120 mg of starch was dissolved in 20 mL of UDMSO solution (100 mL of 6 mol/L urea combined with 900 mL dimethylsulfoxide) in an Erlenmeyer flask, shaken for 3 h, and stored overnight. After 24 h, the flask was shaken for 1 h in a water bath heated to 90 °C; 1 mL of the solution was transferred to a 100 mL volumetric flask and mixed with 90 mL of distilled water and 2 mL iodine solution (0.2% of iodine in 2% KI solution, *w*/*w*) and filled with water. The absorbance of the solution was measured after 15 min at 635 nm. A blank sample contained 1 mL of UDMSO, 97 mL of water, and 2 mL of iodine solution. The blue value (BV) was expressed using the Formula (1), where A is the absorbance and m is the mass of starch (mg).

BV = a × 10/m(1)

### 3.6. Determination of Weight-Average Molecular Weight

The molecular weight (M_W_) distribution of starches was determined by GPC, as described by Bai et al. [62] with modifications: 120 mg of the sample was weighed into a 100 mL conical flask, 20 mL of DMSO was added and shaken for 3 h at room temperature, and then the samples were left for 24 h at room temperature without stirring. After this time, the samples were heated for 1 h in a water bath at 90 °C and then cooled. A total of 100 µL of sample was transferred to a chromatography vial, 900 µL of HPLC-grade distilled water was added, and M_W_ distribution was analyzed. The conditions of chromatographic analysis are as follows: column—PolySep-GFC-P 1000 LC column 300 × 7.8 mm (Phenomenex Manufactures, Torrance, CA, USA); column temperature—30 °C; eluent—water HPLC; flow—0.4 mL min^−1^; time of analysis—40 min; sample injection—10 µL; and detector RI—40 °C. Standard dextrans (Sigma) with different M_W_s were used for M_W_ calibration.

### 3.7. Content of Non-Polysaccharide Components

The content of protein was estimated according to AOAC [63]. The sample was placed in a Kjeldahl flask. A selenium mixture was added as a catalyst, which was followed by the addition of sulfuric acid. Mineralization was performed until the solution turned light green. The flask was then cooled and its content was diluted by adding small portions of water. The sample was transferred to Parnas-Wagner apparatus and treated with a 33% solution of sodium hydroxide. Distillation was performed for about 15 min, and the condensate was collected in a conical flask filled with 0.05 mol/L sulfuric acid and a few droplets of Tashiro indicator. After distillation, the unbound acid was titrated with 33% of sodium hydroxide. The content of ash in starch was estimated according to AOAC [63]. The starch sample (approximately 2 g) was burned in a quartz crucible over Bunsen burner and placed in a hot muffle furnace (900 °C) for about an hour until all traces of carbon burned out. The total phosphorus in starch was analyzed according to EN ISO 3946 [64]. The sample (0.5 g) was mineralized in a 1:1 *w*/*w* mixture of sulfuric and nitric acids (15 mL). Then the sample was diluted with water and brought to pH 7 with an aqueous solution of 10 mol/L NaOH. The neutral solution was treated with ammonium heptamolybdate (VI) tetrahydrate (4 mL) and 50 g/L ascorbic acid (2 mL). The intensity of the absorption band was recorded at 825 nm.

### 3.8. Water-Binding Capacity and Solubility

The water-binding capacity and solubility at 25, 40, 60, 75, and 95 °C were determined using a modified gravimetric procedure by Ulfa et al. [65]. Starch samples (1 g d.m.) were suspended in 70 mL of distilled water and heated at 25, 40, 60, 75, and 95 °C, respectively, for 30 min on agitation by mechanical stirrer type CAT R100CT (Zipperer GmbH, Ballrechten-Dottingen, Germany) (200 rpm). The samples were then centrifuged for 10 min at 1050× *g* (MPW350, Warsaw, Poland). The supernatant was dried in dryer type SML Zalmed (Warsaw, Poland) and used for the calculation of solubility, and solid remnants were the basis for a gravimetric determination of water-binding capacity. Determinations were run in triplicate.

### 3.9. Gelatinization Measurements

The gelatinization properties of starch were determined by differential scanning calorimetry (DSC) analysis, as previously described by Kapuśniak et al. [66], with some modifications. For this purpose, a MICRO DSC III differential scanning calorimeter from Setaram Instrumentation (Caluire-et-Cuire, France) was used. Triplicate starch samples (approximately 40 mg) were weighed in stainless steel, high-pressure-type “batch” cells at a water/starch ratio of 70:30 (*w*/*w*). The samples were heated from 18 to 120 °C at 3 °C/min and then cooled to 20 °C. The onset (*To*), peak (*Tp*), and conclusion (*Tc*) temperatures; gelatinization temperature range *Tc* − *To*; and enthalpy change (Δ*H*) expressed in J/g dry starch) were calculated from thermograms.

### 3.10. Retrogradation Measurement

The enthalpy of retrogradation (Δ*Hr*), starting (*To*), final temperature of transformation (*Tc*), and temperature of maximum peak (*Tp*) were estimated using MICRO DSC III differential scanning calorimeter from Setaram Instrumentation (Caluire-et-Cuire, France). The analysis was carried out within the temperature range of 18 to 120 °C at 3 °C/min. After heating, the samples were stored for 24, 48, and 72 h at 8 °C. Afterwards, the samples were re-heated up to 120 °C with the same heating rate in order to melt the crystals formed during recrystallization.

### 3.11. Degree of Retrogradation

The degree of retrogradation of the 1% water solution at 20, 8, and −20 °C was calculated using the below equation. The Whistler method [51] is based on the absorbance of the iodine–starch complex formed by amylose from a supernatant after centrifugation at point zero and also after 24, 48, and 72 h of storage. Spectrophotometric measurements were carried out in a spectrophotometer (Helios Gamma, 100–240, Runcorn, UK) at 635 nm. The degree of retrogradation was calculated using the following equation, where A is the absorbance of the stored solution and A_0_ is the absorbance at time zero:

DR = 100% − A/A_0_ × 100% (2)

### 3.12. Rheological Measurements

Isolated samples of apple starch in the form of 5% aqueous suspensions were gelatinized at 95 °C for 90 min. After the gelatinization process was completed, the samples were cooled for 60 min. The gelatinized starch samples were then left at an ambient temperature of 25 °C for a further 60 min to remove any air bubbles. After this time, the starch paste samples were placed in the measuring system of the rotational rheometer and left for an additional 30 min at a constant temperature of 50 °C to reach thermal and mechanical equilibrium. The rheological properties of the tested apple starches were determined using a Physica MCR 301 rotational rheometer by Anton Paar (Tokyo, Japan) in a cone-plate measuring system with a cone diameter of 50 mm, an angle of inclination of 1°, and a distance between the measuring elements, i.e., a cone and a plate of 0.048 mm.

Rheological tests were carried out to measure the viscous and viscoelastic properties of the tested apple starch samples.

### 3.13. Measurement of Viscous Properties

Basic studies of the viscous properties were performed in the controlled shear rate mode. The flow curves were determined by measuring the shear stress as a function of the shear rate during three successive stages:

- Increasing shear rate from 0.1 s^−1^ to 100 s^−1^ for 300 s.
- Constant shear rate 100 s^−1^ for 60 s.
- Decreasing shear rate from 100 s^−1^ to 0.1 s^−1^ for 300 s.

The measurements were carried out in triplicate, and the final result is their arithmetic mean. The following rheological models were used to describe the flow curves obtained as a result of rheometric measurements [67]:

- Ostwald de Waele model.

(3) $$\tau =k\cdot \gamma^{n}$$

- Herschel–Bulkley model.

(4) $$\tau =\tau_{y}+k\cdot \gamma^{n}$$

- Casson model.

(5) τ12=τy12+k·γ12

where *τ* is the shear stress (Pa), *γ* is the shear rate (s^−1^), *n* is the flow characteristic index (-), *k* is the consistency coefficient (Pa·s*^n^*), and *τ_y_* is the yield point (Pa).

In order to assess the correctness of the description of the experimental data with the model Equations (3)–(5), a statistical evaluation of the fit of the model curves to the experimental curves was carried out in relation to the shear stress value *τ*. This assessment was made by estimating the modeling efficiency of *R^2^* and the root mean square error of RMSE:

(6) $$R^{2}=\frac{\sum_{i=1}^{N}{\left(\tau_{exp}-\tau_{\exp sr}\right)}^{2}-\sum_{i=1}^{N}{\left(\tau_{mod}-\tau_{exp}\right)}^{2}}{\sum_{i=1}^{N}{\left(\tau_{exp}-\tau_{\exp sr}\right)}^{2}}$$

(7) RMSE=1N∑i=1N(τexp−τmod)212

where *N* is the number of experimental points (-), *τ_exp_* is the experimental shear stress (Pa), *τ_exp sr_* is the average value of experimental shear stress (Pa), and *τ_mod_* is the theoretical value of shear stress from rheological model (Pa).

### 3.14. Measurement of Viscoelastic Properties

The study of viscoelastic properties was carried out in dynamic conditions in the controlled strain mode. The mechanical spectra were determined by measuring the values of the storage modulus *G*′ and the loss modulus *G*″. The tests were carried out in a wide range of oscillation frequencies *ω* from 0.1 rad/s to 100 rad/s, taking 10 measurement points for each tenth decade. The value of relative strain for the tested samples of apple starch was determined in previous studies on the range of linear viscoelasticity; for SP, the relative strain value was 0.25%, and for SO, it was 0.1%. The measurements were made in three repetitions, and the final result is their arithmetic mean.

Based on the carried out rheometric measurements, the following parameters were determined [68]:

- The equilibrium modulus, i.e., the modulus of elasticity in a steady state, *G_e_*, is responsible for the total elasticity of the medium:

(8) $$G_{e}=\lim_{\omega \to 0}G^{\prime }$$

- Viscoelastic plateau modulus *G_N_*^0^ is responsible for cross-linking of the structure, and its high values also indicate the possibility of slowing down the aging effects of the medium over time. For polydisperse media—such as gelatinized apple starches—it is determined by the following relationship:

(9) $${G_{N}}^{0}=\frac{4}{\pi }\int_{\omega_{min}}^{\omega_{max}}G^{\prime \prime }\left(\omega \right)d\ln\left(\omega \right)$$

- Newtonian viscosity in conditions of steady flow *η*_0_ characterizes the flow capacity of a set of elements closed with a minimum number of nodes of the biopolymer network that are capable of individual movement:

(10) $$\eta_{0}=\lim_{\omega \to 0}\frac{G^{\prime \prime }}{{\left(\omega \right)}^{2}}$$

These three basic parameters, obtained directly from rheometric measurements, make it possible to additionally determine the following:

- The equilibrium compliance *J_e_*, which is a measure of the energy stored during a steady state of the biopolymer under low stress conditions:

(11) $$J_{e}=\left({\frac{1}{G}}_{e}\right)$$

- Plateau compliance *J_N_*^0^, representing the strength with which the entanglements of the biopolymer network suppress all kinds of long-range configuration rearrangements:

(12) $$J_{N}^{0}=\left(\frac{1}{G_{N}^{0}}\right)$$

- Weighted average relaxation time *τ_m_* equated with the longest relaxation time:

(13) $$\tau_{m}=J_{e}\cdot \eta_{0}$$

- Numerically average relaxation time *τ*_0_ equated with the shortest relaxation time:

(14) $$\tau_{0}=\frac{\eta_{0}}{{G_{N}}^{0}}$$

- Cross-linking density of the structure *ω*_0_, defining at the same time the end of the viscoelastic area of the plateau:

(15) $$\omega_{0}=\frac{1}{\tau_{0}}$$

- Dimensionless width of the viscoelastic plateau *L*, combining fast and slow dissipation processes, determining the degree of biopolymer polydispersion:

(16) $$L=\frac{\tau_{m}}{\tau_{0}}$$

- Average molecular weight by entanglement *M_e_*, which is the average molecular weight between the topological bonds of the biopolymer network, resulting from the physical (mechanical) entanglement of long biopolymer chains:

(17) $$M_{e}=\frac{\rho_{k}\cdot R\cdot T}{G_{N}^{0}}$$

- Average molecular weight at *M_c_* cross-linking, which is the average molecular weight of biopolymer chains between successive nodes of the network, which can be chemical cross-links, crystalline regions, and even polymer complexes:

(18) $$M_{c}=\frac{\rho_{k}\cdot R\cdot T}{G_{e}}$$

where *ρk* is the density of starch pastes, *R* is the universal gas constant, and *T* is the starch pastes temperature during rheological tests.

- Mesh size of the resulting network ξ, which is one of the characteristic linear dimensions of the created network of viscoelastic material:

(19) ξ=kB·TGN013

where *k_B_* is the Boltzman constant

In addition, the differences in the structure of the pastes of two samples of apple starch, SP and SO, were presented in the form of the so-called reduced curves in the coordinate system *G*′/*G_N_*^0^ = f(*ω*/*ω*_0_) and *G*″/*G_N_*^0^ = f(*ω*/*ω*_0_), evaluating the superposition of the obtained experimental curves. This allows the assessment of the variation in the proportion of components that shape the viscous and elastic properties of the apple starches tested. The lack of superposition of reduced curves indicates the differentiation of the scale of dissipation phenomena within fast or slow dissipation processes, while the full superposition of reduced curves indicates a uniform type of the created structure.

### 3.15. Statistical Analysis

The data were subjected to a single-factor analysis of variance, and the differences between the averages were determined using Duncan’s post hoc test at a significance level of 0.05. The relationships between the analyzed parameters were evaluated using the v Pearson correlation coefficients. The calculations were performed using Statistica 11.0 (StatSoft Inc., Tulsa, OK, USA).

## 4. Conclusions

The starches tested differed in the content of analyzed components and granularity, leading to differences in hydration and thermal properties. SO was characterized by a higher amount of protein (by 10%), ash (49%), and phosphorus (17%) in relation to SP. The water-binding capacity of these two starches was identical from 25 to 65 °C, while at temperatures above 75 °C, SO starch showed higher values by about 20% compared to SP starch. Generally, the solubility of SO was nearly two times higher than that of SP, except at 95 °C, at which the solubility of both tested starches was identical. The enthalpy of gelatinization was 40% higher in the SO variety than in SP, although both starches tested showed a low tendency to retrograde. The weight-average molecular mass of the analyzed starches from both apples was the same.

Each of the tested starches isolated from unripe apples forms gels that are characterized by the predominance of elastic properties over viscous ones. Gels obtained from SP are soft gels with an internal structure resistant to external forces. This was proved by the lower value of Newtonian viscosity under the conditions of steady flow *η*_0_ (637 Pa·s) than in the case of SO, with the value of plateau compliance *J_N_*^0^ higher than in the case of SO. In addition, SP gels are characterized by about 8% greater energy storage capacity (higher value of equilibrium compliance *J_e_*), which in turn, with a higher value of plateau compliance *J_N_*^0^ than in SO, indicates that in SP gels there are such network entanglements that suppress all kinds of long-range configuration rearrangements. All this together leads to the conclusion that gels obtained from SP are characterized by a more stable and resistant internal structure to external forces, including shear forces, and they also have a high ability to dampen mechanical oscillations. SO gels belong to hard gels, characterized by high stiffness, which is indicated primarily by the high value of Newtonian viscosity *η*_0_—70% higher than the value for the SP. The higher stiffness of the gels obtained from the SO is also evidenced by the high value of the viscoelastic module of the plateau *G_N_*^0^—44% higher than the value for gels obtained from the SP. The high value of the *G_N_*^0^ module indicates, however, that the aging processes in time are slower in apple gels of the SO. The differences in the nature of the gels obtained from both varieties of apples, especially the differences in the stability of their internal structure, are also evidenced by the width of the viscoelastic plateau *L*, which is a measure of polydispersity. SO has as much as 33% higher polydispersity, which probably disfavors this variety with regard to the internal structure stability of the gels formed.

## Figures and Tables

**Table 1:** Chemical composition and granularity characteristics of the tested apple starches.

Starch	Chemical Composition and the Weight-Average Molecular Weight (M_w_)					
	Amylose (%)	Protein (%) × 6.25	Lipids (%)	Ash (%)	Phosphorus (%)	M_w_ (g/mol)
SO	15.5 ^a^ ± 0.5	0.567 ^b^ ± 0.06	1.20 ^a^ ± 0.02	0.76 ^b^ ± 0.01	0.108 ^b^ ± 0.001	1.68 × 10^8^
SP	16.3 ^a^ ± 0.1	0.514 ^a^ ± 0.04	1.21 ^a^ ± 0.01	0.51 ^a^ ± 0.01	0.092 ^a^ ± 0.001	1.64 × 10^8^
	Granularity					
	Average diameter (µm)	Min (1%) (µm)	Max (1%) (µm)	<30 µm (%)	30–70 µm (%)	>70 µm (%)
SO	6.4	0.4	132.7	67.3	20.4	12.3
SP	5.2	0.4	100.3	75.2	16.2	8.6

SO—starch from the Oliwka variety; SP—starch from the Pyros variety. Data are presented as the mean value of three replicates ± standard deviation. The mean values signed with the same letters in particular columns are non-significantly different at a 0.05 level of confidence.

**Table 2:** Thermal characteristics and temperature-dependent hydration properties of the tested apple starches.

Starch	Thermal Characteristics				
	Δ*H* (J/g d.m.)	*To* (°C)	*Tp* (°C)	*Tc* (°C)	*Tc* − *To* (°C)
SO	7.59 ^b^ ± 0.12	60.50 ^a^ ± 0.01	64.19 ^a^ ± 0.02	71.31 ^b^ ± 0.01	10.81 ^b^ ± 0.01
SP	5.44 ^a^ ± 0.19	60.88 ^b^ ± 0.01	64.94 ^b^ ± 0.00	71.13 ^a^ ± 0.03	10.25 ^a^ ± 0.04
Temperature					
	25 °C	40 °C	60 °C	75 °C	95 °C
	Solubility S (% d.m.)				
SO	1.65 ^b^ ± 0.04	2.03 ^b^ ± 0.11	2.33 ^b^ ± 0.10 ^b^	3.94 ^b^ ± 0.46	6.98 ^a^ ± 0.85
SP	0.68 ^a^ ± 0.01	0.94 ^a^ ± 0.04	0.95 ^a^ ± 0.06 ^a^	2.20 ^a^ ± 0.08	6.86 ^a^ ± 0.04
	Water-binding capacity WBC (g water/g starch d.m.)				
SO	1.22 ^a^ ± 0.01	1.39 ^a^ ± 0.03	1.49 ^a^ ± 0.00	5.48 ^b^ ± 0.00	8.00 ^b^ ± 0.69
SP	1.25 ^a^ ± 0.04	1.33 ^a^ ± 0.05	1.56 ^a^ ± 0.02	4.51 ^a^ ± 0.26	6.64 ^a^ ± 0.18

Data are presented as mean value of three replicates ± standard deviation. Mean values signed the same letters in particular columns are non-significant different at a 0.05 level of confidence.

**Table 3:** Retrogradation of the tested apple starches.

Retrogradation by DSC					
Starch	Time of Storage (h)	*To* (°C)	*Tp* (°C)	*Tc* (°C)	Δ*Hr* (J/g d.m.)
SO	24	43.25 ^a^ ± 0.01	49.00 ^a^ ± 0.02	52.18 ^a^ ± 0.03	0.71 ^b^ ± 0.03
	48	51.00 ^b^ ± 0.03	47.36 ^a^ ± 0.01	57.30 ^a^ ± 0.00	0.83 ^b^ ± 0.01
	72	45.03 ^a^ ± 0.02	51.73 ^b^ ± 0.03	59.61 ^a^ ± 0.01	0.91 ^b^ ± 0.02
SP	24	46.28 ^b^ ± 0.01	52.10 ^b^ ± 0.00	61.09 ^b^ ± 0.02	0.59 ^a^ ± 0.05
	48	41.90 ^a^ ± 0.00	50.04 ^b^ ± 0.02	58.70 ^b^ ± 0.01	0.68 ^a^ ± 0.04
	72	49.11 ^b^ ± 0.01	51.67 ^a^ ± 0.00	63.43 ^b^ ± 0.01	0.79 ^a^ ± 0.02
Degree of retrogradation (%)					
		Temperature of storage			
		20 °C	8 °C	−20 °C	
SO	24	3.07 ^b^ ± 0.07	4.44 ^b^ ± 0.08	49.51 ^b^ ± 0.54	
	48	4.94 ^b^ ± 0.00	6.82 ^b^ ± 0.06	-	
	72	5.83 ^b^ ± 0.07	8.63 ^b^ ± 0.06	-	
SP	24	2.59 ^a^ ± 0.05	3.91 ^a^ ± 0.02	42.58 ^a^ ± 0.27	
	48	3.81 ^a^ ± 0.10	4.53 ^a^ ± 0.05	-	
	72	4.59 ^a^ ± 0.03	7.59 ^a^ ± 0.00	-	

Data are presented as the mean value of two replicates ± standard deviation. The mean values signed with the same letters in particular columns are non-significantly different at a 0.05 level of confidence, separately for each time of storage (24, 48, and 72 h) and for each kind of starch.

**Table 4:** Rheological parameters of models described by Equations (3)–(5).

		Ostwald de Waele Model								
Starch	Upper Curve					Lower Curve				
	*τ_y_*	*n*	*k*	*R^2^*	RMSE	*τ_y_*	*n*	*k*	*R^2^*	RMSE
SO	-	0.176 ± 0.001	11.177 ± 0.160	0.998	0.912	-	0.361 ± 0.002	4.155 ± 0.106	0.991	1.071
SP	-	0.308 ± 0.003	4.269 ± 0.081	0.993	1.060	-	0.303 ± 0.001	3.915 ± 0.074	0.983	1.282
		Herschel–Bulkley Model								
	Upper Curve					Lower Curve				
	*τ_y_*	*n*	*k*	*R^2^*	RMSE	*τ_y_*	*n*	*k*	*R^2^*	RMSE
SO	1.947 ± 0.016	0.499 ± 0.004	2.046 ± 0.007	0.998	0.470	1.832 ± 0.011	0.497 ± 0.002	1.731 ± 0.018	0.998	0.432
SP	5.230 ± 0.033	0.292 ± 0.001	5.622 ± 0.026	0.998	0.600	1.240 ± 0.008	0.463 ± 0.002	2.689 ± 0.021	0.998	0.564
		Casson Model								
	Upper Curve					Lower Curve				
	*τ_y_*	*n*	*k*	*R^2^*	RMSE	*τ_y_*	*n*	*k*	*R^2^*	RMSE
SO	8.748 ± 0.114	-	0.271 ± 0.002	0.990	1.432	2.251 ± 0.010	-	0.416 ± 0.005	0.988	1.557
SP	2.575 ± 0.025	-	0.346 ± 0.003	0.989	1.056	2.373 ± 0.013	-	0.327 ± 0.002	0.997	0.679

Data are presented as the mean value of three replicates ± standard deviation.

## Data Availability

Data are contained within the article.
